# Type 3A Symbrachydactyly in a Newborn Female: A Case Report

**DOI:** 10.7759/cureus.41958

**Published:** 2023-07-16

**Authors:** Noah Gabor, Travis Satnarine, Laurent King, Karen Chen Chen, Patricia Alvarez

**Affiliations:** 1 Pediatrics, University of Miami Miller School of Medicine, Jackson Memorial Hospital, Miami, Florida, USA

**Keywords:** subclavian artery dysgenesis, poland syndrome, hand deformity, congenital abnormality, congenital, symbrachydactyly

## Abstract

Symbrachydactyly is a complex and rare congenital hand deformity characterized by missing or underdeveloped fingers and rudimentary digit nubbins. This case report focuses on a newborn female with type 3A symbrachydactyly, highlighting the unique clinical presentation, diagnostic assessment, and initial management approach. The rarity of this condition underscores the need for sharing cases to enhance understanding and treatment strategies. Various classification systems exist, contributing to the challenge of accurately categorizing symbrachydactyly. Surgical interventions play a crucial role in restoring hand function and appearance, with treatment choices tailored to individual evaluation and goals. Early surgical intervention is often necessary to improve outcomes, and nonvascularized toe phalangeal transfers have shown promising results. Further research is required to uncover the underlying cause and pathogenesis of symbrachydactyly, enabling more targeted and effective treatment approaches. This case report contributes to the existing knowledge and management of this uncommon congenital anomaly, emphasizing the importance of sharing and studying such cases for improved patient care.

## Introduction

Symbrachydactyly is a congenital abnormality of the embryonic limbs that affects the growth of the hand plate, nail plate, bone, and cartilage [1]. With only 0.6 instances per 10,000 live births, it is extremely rare [2]. The absence of finger development and the presence of rudimentary digit nubbins characterize this congenital hand deformity. Symbrachydactyly is usually an isolated condition, but it can be associated with Poland syndrome, which can affect the upper extremity, in which hypoplasia or absence of the pectoralis major occurs with additional variable abnormalities [1]. A hand that is smaller than the opposite side, missing or undeveloped fingers, and rudimentary nubbins made of ectodermal tissue are often noted in this disorder [1]. The underlying cause of symbrachydactyly is generally unknown, but subclavian artery dysgenesis is the leading hypothesis [3]. Mild forms may not require surgical correction. Surgical techniques are aimed at restoring function and appearance and include distraction lengthening, web-syndactyly release, and toe-phalanx transfer [4]. The choice of treatment is crucial, considering practical, scientifically proven cosmetic and functional benefits [2].

In this instance, we talk about a baby girl who has type 3A symbrachydactyly. By detailing our approach and discussing this case, we hope to improve other clinicians' understanding of this uncommon anomaly, its presentations, and the available treatments.

## Case presentation

A healthy newborn female was born at 38 weeks and 5 days to a 20-year-old G2 P1011 woman, who had a history of scant prenatal care and no significant medical or surgical history. The patient was born via spontaneous vaginal delivery with no complications. There is neither a family history of genetic syndromes nor congenital malformations. Obstetric ultrasound at 27 weeks and 4 days revealed that the left hand appeared to be missing digits, with the repeat ultrasound at 35 weeks and 1 day not clearly showing the left hand due to fetal crowding.

**Figure 1:**
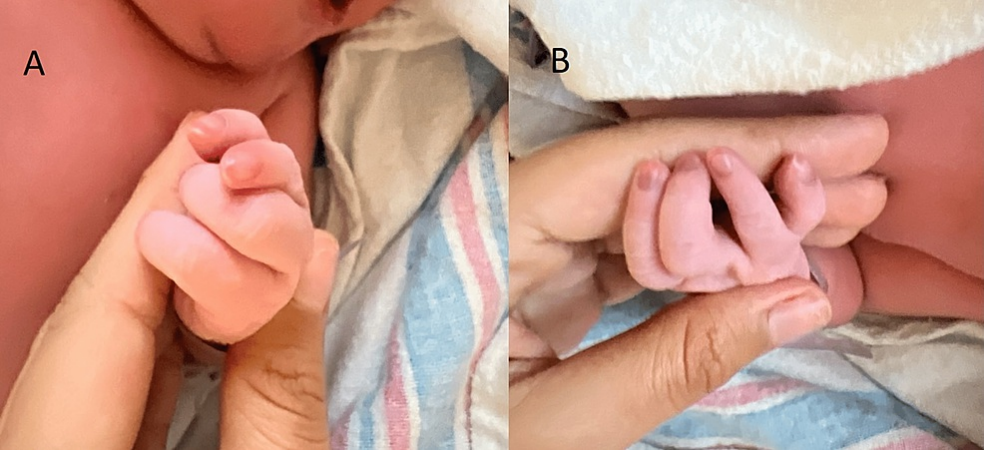
A and B: symbrachydactyly of the left hand

Upon physical examination, the patient was noted to have a left-hand deformity, whereby she was missing the ulnar digit, there was an ulnar deviation of the left fourth digit, and there was decreased/absent webspace between the left first and second digits, as seen in Figure 1. The right hand was completely normal in appearance. The patient was in stable condition, with normal vital signs and no acute distress. The patient was able to spontaneously move all joints of the fingers present; all the skin at the extremities was intact; and there was brisk capillary refill in all fingers.

**Figure 2:**
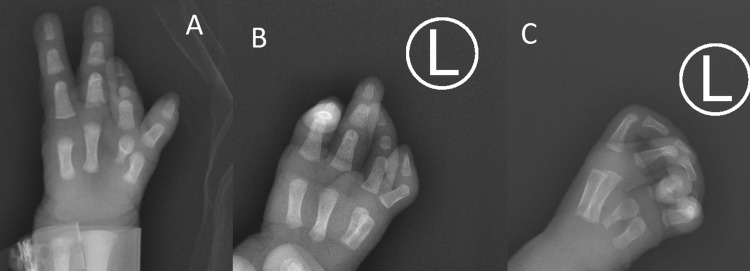
Initial X-ray of the left hand A: X-ray of the left hand taken shortly after birth. B and C: X-ray of the left hand taken at approximately one month of life.

The diagnostic assessment involved physical examination and imaging. The initial X-ray was performed shortly after birth. The findings included the absence of the ulnar digit and the fifth metacarpal bone. The second finger displayed a hypoplastic middle phalanx, indicating underdevelopment and foreshortening. Only three fingers were identified on the medial side of the thumb, as seen in Figure 2A. During the follow-up X-ray, the left hand's bone structure was assessed again. The one-month interval between the X-rays provided an opportunity to evaluate any changes or progression in the left-hand deformity. However, the findings from the follow-up X-ray remained consistent with the initial diagnosis of symbrachydactyly, as seen in Figure 2B* *and Figure 2C.

The patient has been evaluated outpatient by pediatric orthopedic surgery, who are planning for regular follow-up, with a repeat visit scheduled in six months, with repeat radiographs of the left hand at that time, and an aim for surgical reconstruction in the future.

## Discussion

Symbrachydactyly is a complex congenital hand malformation that exhibits various manifestations and poses challenges in its management. The rarity of this condition further emphasizes the importance of sharing cases and discussing treatment strategies to enhance medical professionals' understanding and improve patient care.

Different criteria can be used to categorize symbrachydactyly. Symbrachydactyly is classified by one classification scheme, the Oberg-Manske-Tonkin (OMT) classification, as a lack of axis development and differentiation of the hand plate or the entire upper limb [5]. Based on the degree of the deformity, Blauth and Gekeler's proposed classification system separates symbrachydactyly into four kinds [6]. A more thorough classification of seven kinds based on morphological and radiographic bone deficit was also described by Yamauchi and Tanabu [2]. The diagnosis of symbrachydactyly has been expanded by some physicians to include other characteristics such as cleft hand, terminal transverse limb abnormalities, and hypoplasia of the thumb and fifth finger [7]. In general, there is disagreement among hand surgeons about how to properly classify symbrachydactyly [6].

Based on Foucher’s classification, the presence of a normal thumb differentiates our diagnosis of type 3A symbrachydactyly from other similarly presenting conditions that lack the presence of an ulnar digit [2]. These include type 3B symbrachydactyly, which presents with a hypoplastic thumb, and types 4A and 4B, which both present with the absence of the thumb [2]. The other subtypes of type 1 and types 2A, 2B, and 2C all present with the presence of a full or hypoplastic ulnar digit [2]. This differentiation is crucial, as it influences the choice of treatment and prognosis [2].

The management of symbrachydactyly involves a surgical intervention to restore hand function and appearance [8]. Surgical options include procedures such as thumb web creation, thumb lengthening, opposition post creation, syndactyly release, web-space deepening, joint fusion, nubbin excision, phalanx lengthening, toe phalanx transfer, and neurovascular transfer of a toe or toes [4]. The choice of surgical intervention should be based on a comprehensive evaluation of the child's functional abilities and hand appearance, with the surgeons discussing the goals and expectations of the surgery with the parents to ensure the most effective outcome [9]. Early surgical intervention is often necessary to improve hand function and quality of life [8].

The prognosis of symbrachydactyly depends on the severity of the condition and the chosen surgical intervention. Nonvascularized toe phalangeal transfers can also be a viable treatment option for select cases of symbrachydactyly, resulting in a functional hand with mobile metacarpophalangeal joints [10]. Long-term outcomes of free nonvascularized toe phalanx transfer have shown promising results in reconstructing symbrachydactyly hands in children [11]. Further studies are needed to better understand the functional changes associated with different interventions and to improve treatment decisions.

It is worth noting that the underlying cause of symbrachydactyly remains largely unknown, and further research is needed to elucidate the etiology and pathogenesis of this condition, which would aid in developing more targeted and effective treatment approaches.

## Conclusions

Type 3A symbrachydactyly in a newborn female is rare, as represented by our case. Accurate diagnosis and subtype distinction are emphasized. Mild cases may not require surgical intervention. Surgical techniques, such as distraction lengthening, web-syndactyly release, and toe-phalanx transfer, are aimed at improving function and appearance. Sharing and studying such examples improves patient care and management by increasing knowledge of this rare congenital condition.
